# Controllable Majorana vortex states in iron-based superconducting nanowires

**DOI:** 10.1093/nsr/nwac095

**Published:** 2022-05-17

**Authors:** Chuang Li, Xun-Jiang Luo, Li Chen, Dong E Liu, Fu-Chun Zhang, Xin Liu

**Affiliations:** School of Physics and Institute for Quantum Science and Engineering, Huazhong University of Science and Technology, Wuhan 430074, China; Wuhan National High Magnetic Field Center and Hubei Key Laboratory of Gravitation and Quantum Physics, Wuhan 430074, China; School of Physics and Institute for Quantum Science and Engineering, Huazhong University of Science and Technology, Wuhan 430074, China; Wuhan National High Magnetic Field Center and Hubei Key Laboratory of Gravitation and Quantum Physics, Wuhan 430074, China; School of Physics and Institute for Quantum Science and Engineering, Huazhong University of Science and Technology, Wuhan 430074, China; Wuhan National High Magnetic Field Center and Hubei Key Laboratory of Gravitation and Quantum Physics, Wuhan 430074, China; State Key Laboratory of Low Dimensional Quantum Physics, Department of Physics, Tsinghua University, Beijing 100084, China; Kavli Institute for Theoretical Sciences and CAS Center for Excellence in Topological Quantum Computing, University of Chinese Academy of Sciences, Beijing 100190, China; Collaborative Innovation Center of Advanced Microstructures, Nanjing University, Nanjing 210093, China; School of Physics and Institute for Quantum Science and Engineering, Huazhong University of Science and Technology, Wuhan 430074, China; Wuhan National High Magnetic Field Center and Hubei Key Laboratory of Gravitation and Quantum Physics, Wuhan 430074, China

**Keywords:** iron-based superconductor, topological phase transition, superconducting vortex, Majorana zero modes, nanowire

## Abstract

To reveal the non-Abelian braiding statistics of Majorana zero modes (MZMs), it is crucial to design a Majorana platform, in which MZMs can be easily manipulated in a broad topological nontrivial parameter space. This is also an essential step to confirm their existence. In this study, we propose an iron-based superconducting nanowire system with Majorana vortex states to satisfy desirable conditions. This system has a radius-induced topological phase transition, giving a lower bound for the nanowire radius. In the topological phase, the iron-based superconducting nanowires have only one pair of MZMs over a wide range of radii, chemical potential and external magnetic fields. The wave function of MZMs has a sizable distribution at the side edge of the nanowires. This property enables the control of the interaction of MZMs in neighboring vortex nanowires and paves the way for Majorana fusion and braiding.

## Teaser

The iron-based superconducting nanowire is proposed to be a novel desired platform for validating Majorana modes and non-Abelian braiding statistics.

## INTRODUCTION

Majorana zero modes (MZMs) have attracted considerable theoretical and experimental attention given their non-Abelian statistics. The physical realization of MZMs can be roughly classified into two: end MZMs in one-dimensional (1D) systems [1–4] and vortex MZMs in two dimensions [5–7]. In pioneering studies, the superconducting proximity effect plays a paramount role in developing experimentally realizable platforms, e.g. superconductor (SC)/semiconductor (Sm) hybrid nanowires [8–11] and Fe chains growing on SCs [12,13] for 1D cases and SC/topological-insulator (SC/TI) heterostructures [14–16] for 2D cases. Despite the impressive progress in the epitaxial growth of SCs [17], the requirement of the ultraclean heterogeneous interface encounters various difficulties in fabrication techniques and experimental measurements [18]. Recent studies have revealed that iron-based superconducting materials simultaneously possess superconductivity and topological energy band structure and can hence support vortex MZMs without fabricating complex heterostructures. Meanwhile, the small Fermi energy and large superconducting gap in iron-based SC enable MZMs to be distinguished from conventional Caroli–de Gennes–Matricon states [19]. These offer great advantages for the experimental realization and detection of MZMs [19–27].

Moreover, observing the zero-bias peak is a necessary but not sufficient condition for detecting MZMs. Perhaps, only the experimental observation of MZM fusion behaviors and braiding statistics can provide the smoking gun signature. Therefore, implementing a Majorana platform optimized for MZM fusion and braiding is the key to the next milestone. Such an optimized platform should satisfy at least three conditions: (1) a broad parameter range to support well-defined topological degeneracy, (2) an efficient control scheme for MZMs and (3) a high fidelity and easy readout scheme. For the vortex MZMs, satisfying the first condition requires precise control of the number of vortex lines in the system, although parameter fine-tuning is unnecessary. The second condition is related to braiding MZMs, which can be intuitively achieved in real space [28], and their physical implementation is challenging for existing experimental techniques. The promising schemes to perform braiding need to control the neighboring Majorana couplings [29–33]. Finally, satisfying the third condition also presupposes a reliable control of Majorana couplings. To the best of our knowledge, there is no detailed physical scheme in the vortex MZM platforms satisfying these three conditions.

**Figure 1.**
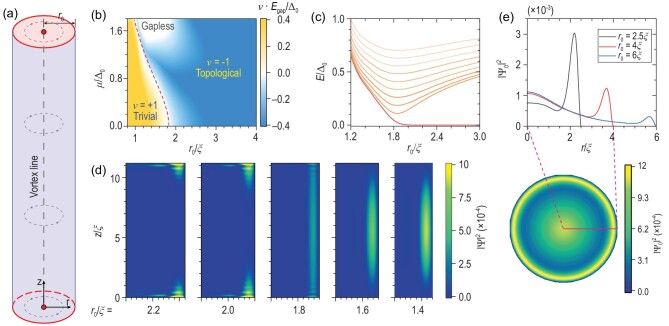
The size effect-induced TPT in the iron-based superconducting nanowire vortex system described by Equation (1). (a) The sketch of an iron-based superconducting nanowire system. (b) The product of the topological invariant ν and the system gap amplitude *E*_gap_ as a function of the nanowire radius *r*_0_ and chemical potential μ. (c) The variation in the ten lowest positive eigenenergies near the TPT at μ = 0 with open boundary in the *z* direction. (d) The probability density |Ψ|^2^(*r, z*) of the lowest-energy states in the nanowires with *r*_0_ = 2.2ξ, …, 1.4ξ. (e) The distribution of the MZMs' probability density |Ψ_0_|^2^ on the circular surface before the TPT. The parameters used in the calculations are *M* = 20 meV, *A* = 30 meV nm, *A*_3_ = 2.7 meV nm, *B* = −31 meV nm^2^, *B*_3_ = 4.7 meV nm^2^, Δ_0_ = 1.8 meV so that ξ ≈ 5.3 nm, and 60 layers spaced by 0.6 nm in the *z* direction.

In this study, we propose an iron-based superconducting nanowire setup [considering the (001) direction as an example in Fig. 1(a)] to satisfy the three conditions. Because the repulsive interaction exists among vortices with finite distances, there is only a single vortex with one pair of MZMs in a wide range of magnetic fields and radii, causing unambiguous two-fold degenerate ground states. Besides the well-known topological phase transition (TPT) from varying the chemical potential μ [34], we found an additional TPT by tuning the radius *r*_0_ of the iron-based superconducting nanowire. Interestingly, unlike the well-known case, the transition occurs at the chemical potential within the bulk gap. This TPT indicates a lower bound for the nanowire radius that can support MZMs. Moreover, there is a radius range within the topologically nontrivial phase, where the Majorana wave function is distributed with a substantial weight near the vortex center as well as on the nanowire lateral surface. The wave function distribution in the lateral surface allows a gate-tunable coupling between vortex MZMs via their edge contacts. This radius region can be further extended by introducing a local effective Zeeman field (ZF), for example, on the bottom surface where the bottom MZM does not disappear but moves to the bottom edge. This structure is topologically equivalent to a 2D topological SC possessing a single vortex. Notably, one iron-based superconducting nanowire has length *l*_0_ and radius *r*_0_, which allows for proper separation between the bottom and top MZMs while sufficiently maintaining a large gap between the MZMs and Bogoliubov quasiparticle states to prevent quantum information leakage. In parallel to the benefit of achieving MZMs without requiring proximity effects, our scheme embodies great advantages to satisfy the conditions of the optimized Majorana platform. First, the geometry of the nanowires will ensure that there are only two MZMs within a certain radius, thereby providing well-defined doubly degenerated ground states in the Majorana vortex system. Second, the edge MZMs are easily controlled. Third, the next step in non-Abelian statistic studies can be performed using braiding schemes developed from the Sm nanowires.

The rest of this article is organized as follows. First, we discuss the TPT due to the radius of the iron-based superconducting nanowires and the spatial distribution of Majorana vortex modes. Second, to obtain tunable MZMs in a wide range of nanowire radii, we induce a local effective ZF on the bottom surface to push the bottom Majorana vortex to the edge. In addition, we investigate the spectral variation and evolution of MZM wave function. Third, we analyze the gate voltage controllable MZM coupling with and without the local ZF. Then, we discuss the repulsion between vortices in the finite-sized nanowire, which allows the number of MZMs and hence the ground-state degeneracy of the nanowire to be regulated by the magnetic field. Finally, we summarize our results and discuss possible experimental challenges.

## TPT AND THE MAJORANA VORTEX WAVE FUNCTION IN AN IRON-BASED SUPERCONDUCTING NANOWIRE

Focusing on the topological band structure near the Fermi surface, the Bogoliubov–de Gennes (BdG) Hamiltonian of the iron-based SC can be expressed as

(1) \begin{eqnarray*} H_{\rm S}= \left(\begin{array}{cc}H_{\rm TI}(\boldsymbol {r}) -\mu &\quad \hat{\Delta }_n(\boldsymbol {r}) \\ \hat{\Delta }_n^{\dagger }(\boldsymbol {r}) &\quad -H_{\rm TI}^*(\boldsymbol {r}) +\mu \end{array}\right), \end{eqnarray*}

in the basis $(C(\boldsymbol {r}),\, C^\dagger (\boldsymbol {r}) )^T$, with $C(\boldsymbol {r})= \left( c_{1\uparrow }(\boldsymbol {r}),\, c_{1\downarrow }(\boldsymbol {r}),\, c_{2\uparrow }(\boldsymbol {r}),\, c_{2\downarrow }(\boldsymbol {r}) \right)^T$ as the electron annihilation operator in real space; subscripts (1,2) and (↑, ↓) indicate the orbital and spin degrees of freedom, respectively, and μ denotes the chemical potential measured from the Dirac point of the surface states. Here, *H*_TI_ is expressed as [24,35,36]

(2) \begin{eqnarray*} H_{\rm TI}(\boldsymbol {k})&=& A \hat{\sigma }_x ( \hat{s}_x\sin {k_x} +\hat{s}_y\sin {k_y} ) \\ &&+\,\hat{\sigma }_z [ M -B ( 4-2\cos {k_x}-2\cos {k_y} ) ] \\ &&+\, A_3 \hat{\sigma }_x \hat{s}_z\sin {k_z} -\hat{\sigma }_z B_3( 2-2\cos {k_z} )\\ \end{eqnarray*}

with *M, A, A*_3_, *B* and *B*_3_ the anisotropic material parameters, and $\hat{\boldsymbol{\sigma }}$ and $\hat{\boldsymbol{s}}$ the Pauli matrices acting on the orbital and spin spaces, respectively. Because the magnetic field in vortices is small, its effect on bands is neglected. The superconducting term is expressed as

(3) \begin{eqnarray*} \hat{\Delta }_n(\boldsymbol {r}) = -i\hat{s}_y\Delta _0 \bigg (\tanh \bigg (\frac{r}{\xi }\bigg )e^{i\varphi }\bigg )^n, \end{eqnarray*}

where *n* = 0 (or 1) for a system with no (or one) vortex, Δ_0_ denotes the amplitude of the bulk superconducting order parameter and ξ = ℏ*v_F_*/(πΔ_0_) denotes the superconducting coherence length with Fermi velocity *v_F_*. For *n* = 1, the chemical potential μ can induce a TPT with the transition above the bulk bandgap [34,36,37]. Remarkably, we found that in the iron-based superconducting nanowires, the size effect, precisely the radius *r*_0_, induces an additional TPT.

We adopt a continuous model in the *x*-*y* plane. Then, the system has the continuous rotational symmetry with angular momentum *j* for *H*_S_. For *n* = 1, we block-diagonalize the Hamiltonian with a quantized number $j\in \mathbb {Z}$ in the *r*-*z* space as (see the online supplementary material for details)

(4) \begin{eqnarray*} &&\!\!\!\!\!\!\mathcal {H}^{(j)}(r,z)\\ &&= \left(\begin{array}{cc}\mathcal {H}_{\rm TI}^{(j)} -\mu &\,\,\,\,\,\, -i\hat{s}_y \Delta _0\tanh ({r}/{\xi }) \\ i\hat{s}_y \Delta _0\tanh ({r}/{\xi }) &\,\,\,\,\,\, -{\mathcal {H}_{\rm TI}^{(-j)}}^* +\mu \end{array}\right),\\ \end{eqnarray*}

where

(5) \begin{eqnarray*} \mathcal {H}_{\rm TI}^{(j)} &=& -iA \hat{\sigma }_x \bigg [ \hat{s}_x \partial _r +\frac{i(j+1/2)}{r} \hat{s}_y +\frac{1}{2r} \hat{s}_x \bigg ] \\ && +\,\hat{\sigma }_z \bigg ( M +B \bigg [ \partial _r^2 +\frac{1}{r}\partial _r\\ && -\,\frac{1}{r^2}\bigg ( j-\frac{\hat{s}_z-1}{2} \bigg )^2 \bigg ] \bigg ) \\ && +\, A_3 \hat{\sigma }_x \hat{s}_z\sin {k_z} -\hat{\sigma }_z B_3( 2-2\cos {k_z}).\\ \end{eqnarray*}

In the rest of this study, we use the calligraphic font to describe the Hamiltonian for a fixed *j*. We performed numerical calculations using the Kwant code [38].

A superconducting system with vortex lines along the *z* direction can be considered a quasi-1D system, which belongs to class D of the Altland–Zirnbauer classification [39]. Notably, the particle-hole symmetry yields $\hat{P}\mathcal {H}^{(j)}\hat{P}^{-1}=-\mathcal {H}^{(-j)}$. Therefore, if the system is fully gapped, its topology is characterized by the $\mathbb {Z}_2$ topological invariant ν [1]:

(6) \begin{eqnarray*} \nu ={\rm sgn}\bigg \lbrace \frac{{\rm Pf}[\mathcal {H}_{\rm Mj}^{(0)}(r_0,k_z=0)]}{{\rm Pf}[\mathcal {H}_{\rm Mj}^{(0)}(r_0,k_z=\pi )]}\bigg \rbrace \end{eqnarray*}

with $\mathcal {H}^{(0)}_{\rm Mj}$ the Hamiltonian $\mathcal {H}^{(j=0)}$ written in the Majorana basis. We plot the product of the topological invariant ν and the system gap amplitude *E*_gap_ as a function of *r*_0_ and μ in Fig. 1(b), explicitly showing a TPT, characterized by the sign change of ν and gap closure. To further understand this TPT, at μ = 0 with an open boundary condition along the *z* direction, we plot the energy spectrum [Fig. 1(c)] and lowest-energy wave function [Fig. 1(d)] with respect to the nanowire radius. The emergence of the zero-energy mode above the critical radius *r_c_* ≈ 1.8ξ ≈ 10 nm indicates a TPT and the MZM. As the radius decreases, the MZM wave function shifts toward the edge [also shown in Fig. 1(e)]. When the nanowire radius crosses the critical radius *r_c_*, the MZMs on the upper and lower surfaces gradually couple through the lateral surface [Fig. 1(d)]. These suggest that the TPT is related to the surface states on the lateral boundary, which can be confirmed by studying the Hamiltonian of the system at the band inversion point *k_z_* = π.

**Figure 2.**
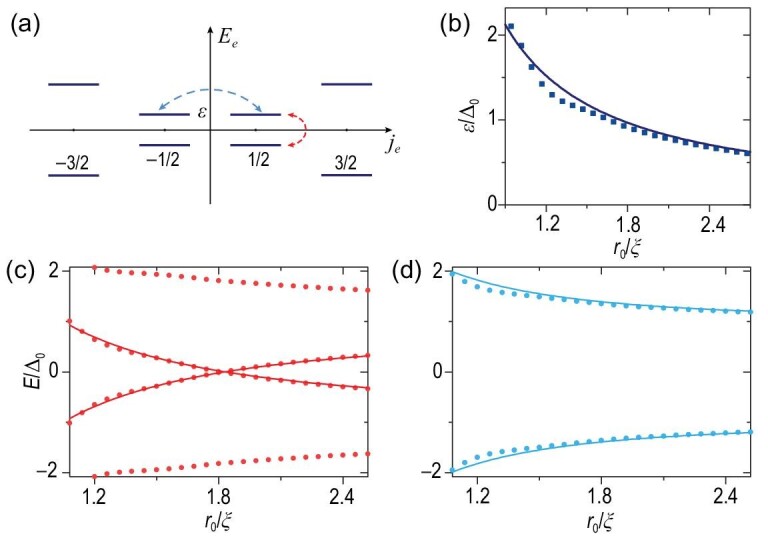
(a) The spectrum illustration of low-energy states of the electron Hamiltonian part *H*_TI_ from Equation (2) with *k_z_* = π. (b) The lowest positive eigenenergy of the TI for *j_e_* = 1/2 and *k_z_* = π. The curve is the approximate energy given by Equation (9). (c),(d) Low energies (dots) of the superconducting nanowire with *k_z_* = π in the presence or absence of a single vortex corresponding to the electron pair indicated by a red or cyan dashed line, respectively, in (a). The curves are approximate analytical energies given by Equations (8) and (10). The TI parameters are the same as those in Fig. 1.

Notably, the corresponding electron Hamiltonian *H*_TI_ of Equation (2) always satisfies time-reversal symmetry; meanwhile, in Equation (1), only the superconducting gap function with one vortex (*n* = 1) breaks the time-reversal symmetry. Thus, the electronic spectrum [Fig. 2(a)] remains time-reversal invariant, say $E_{\!j_e} = E_{-j_e}$, with *j_e_* the electronic angular momentum for *H*_TI_. For *n* = 1, the time-reversal symmetry breaking is reflected in the fact that the Cooper pairs are formed by coupling two electrons with angular momentum (*j_e_*, −*j_e_* + 1) [indicated by the red dashed double arrows in Fig. 2(a)]. Considering *j_e_* = 1/2 as an example, the corresponding BdG Hamiltonian projected to *j_e_* = 1/2 sector states takes the form

(7) \begin{eqnarray*} H_{j_e = {1}/{2}} \approx \left(\begin{array}{cc}\varepsilon (r_0) \hat{s}_z -\mu \hat{s}_0 &\quad - i\hat{s}_y\Delta (r_0) \\ i\hat{s}_y\Delta (r_0) &\quad -\varepsilon (r_0) \hat{s}_z +\mu \hat{s}_0 \end{array}\right) , \\ \end{eqnarray*}

which gives rise to the two lowest eigenenergies

(8) \begin{eqnarray*} E_{\pm }(r_0) = \pm [ \varepsilon (r_0) - \sqrt{\Delta ^2(r_0)+\mu ^2} ] \end{eqnarray*}

with

(9) \begin{eqnarray*} \varepsilon (r_0) = \frac{A }{2r_0}-\frac{B }{2r_0^2} \end{eqnarray*}

the eigenenergy in the electron spectrum [Fig. 2(b)] (see Sec. II within the online supplementary material for details). According to Equation (8), the BdG Hamiltonian of Equation (7) closes its gap at $\varepsilon (r_c) = \sqrt{\Delta ^2(r_c) + \mu ^2}$. In addition, the gap closing in other *j_e_* sectors always occurs an even number of times because of the particle-hole symmetry. Therefore, the sector of *j_e_* = 1/2 determines the TPT. We numerically calculate and plot the four eigenenergies closest to zero as a function of *r*_0_ with red dots when μ = 0 in Fig. 1(c), which agree with our analytical results (the red solid curves) obtained from Equation (8). In particular, ignoring the spatial variation of Δ, the critical point could be simplified as *r_c_* ≈ *A*/(2Δ_0_) = πξ/2. As a comparison, for the superconducting nanowire without vortices (*n* = 0), the electron states will couple their time-reversal partner in SC [indicated by the blue double arrows in Fig. 2(a)], and the energies become

(10) \begin{eqnarray*} E_{\pm }(r_0) = \pm \sqrt{[ \varepsilon (r_0) -\mu ]^2+\Delta _0^2}, \end{eqnarray*}

which is always fully opened by the superconducting gap [Fig. 2(d)], corresponding to no TPT and no topological region in the nanowire.

Notably, near the TPT, the lowest-energy wave functions always distribute at the nanowire boundary, which agrees with the small decay length of the lateral TI surface states of the electron Hamiltonian *l_e_* ≈ 2 (nm) ≪ *r_c_*. Therefore, the energy gap ϵ(*r*_0_) [Equation (9)] and TPT in the superconducting nanowire is not due to the overlap between the TI surface states. As the TPT occurs at the lateral surface, we find in Fig. 1(d) and (e) that, when *r*_0_ is slightly greater than *r_c_*, the MZMs can have a considerable weight on the edge. For example, in Fig. 1(e), when *r*_0_ shrinks to the magnitude of 4ξ, the distribution of MZMs at the edges is larger than that at the center. This finite distribution of MZMs at edges will enable the coupling of two MZMs from parallel nanowires, which we discuss in the section after next.

## LOCAL EFFECTIVE ZF AND EDGE MZMs

For thick iron-based superconducting nanowires or materials with short superconducting coherence length, the MZM wave function is mainly concentrated in the vortex center, which is unfavorable for MZM manipulation. To improve MZM controllability, a local effective ZF is added at the bottom surface $\hat{V}_{\rm Z}(z) = -V_z \delta (z)\hat{\tau }_z\hat{s}_z$ to push the MZMs to the edge. We propose to use an intralayer ferromagnetic and interlayer antiferromagnetic substrate to generate the effective ZF through short-range exchange interactions without introducing the undesired magnetic field [40,41]. Then, the Hamiltonian of the iron-based superconducting nanowires becomes

(11) \begin{eqnarray*} &&\!\!\!\mathcal {H}^{(j)}(r,z)\\ &&= \left(\begin{array}{cc}\mathcal {H}_{\rm TI}^{(j)} +\hat{V}_{\rm Z} -\mu &\quad -i\hat{s}_y \Delta _0\tanh ({r}/{\xi }) \\ i\hat{s}_y \Delta _0\tanh ({r}/{\xi }) &\quad -{\mathcal {H}_{\rm TI}^{(-j)}}^* -\hat{V}_{\rm Z} +\mu \end{array}\right) . \\ \end{eqnarray*}

**Figure 3.**
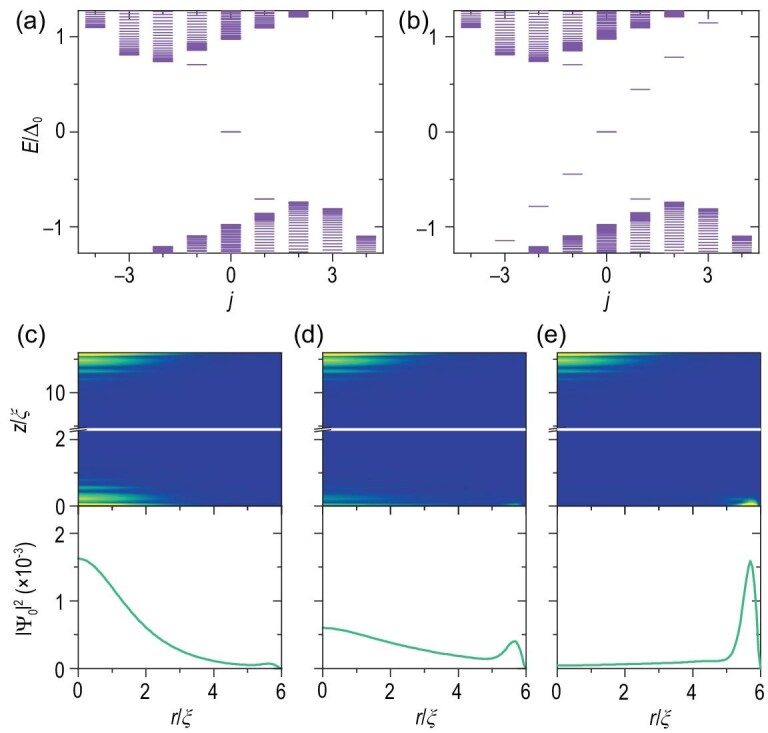
The spectra of the iron-based superconducting nanowire system in Equation (11) with the bottom layer effective ZF (a) *V_z_* = 0 and (b) *V_z_* = 4Δ_0_ at μ = 1.2Δ_0_. (c)–(e) The MZM distribution |Ψ_0_|^2^ near the top and bottom surfaces corresponding to *V_z_* = 0, 2 and 4 (Δ_0_) in sequence. The lower panels show the probability density |Ψ_0_(*r*)|^2^ on the bottom surface. Other parameters are the same as those in Fig. 1.

The local effective ZF changes the surface state energy gap from superconducting dominant to magnetic dominant at the bottom surface. In general, when $\vert V_z \vert > \sqrt{\Delta _0^2 + \mu ^2}$, the superconductivity of this bottom surface will be completely suppressed. In this case, the MZM at the bottom surface will be distributed around the bottom edge, say the boundary between the insulating bottom surface and superconducting lateral surface. Therefore, the original vortex MZM becomes a chiral MZM [6,42,43]. This can be seen from the spectrum and MZM distribution of the nanowires in Fig. 3.

**Figure 4.**
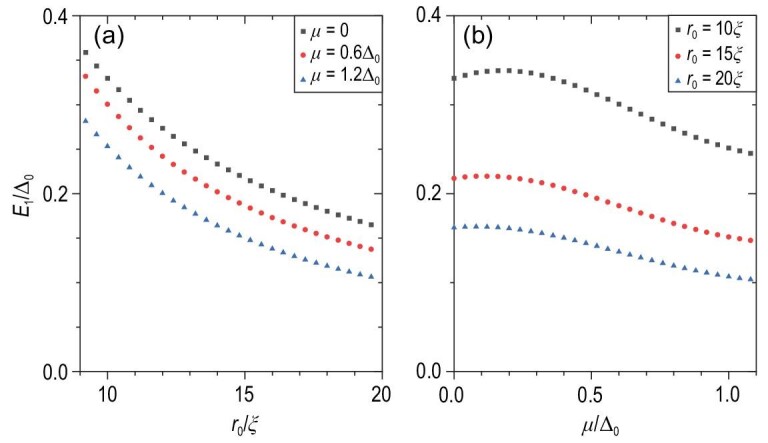
The scatter graphs of the first excited energy *E*_1_ of the Hamiltonian in Equation (11) with respect to (a) the superconducting nanowire’s radius *r*_0_ for different chemical potentials and (b) the chemical potential μ for different radii of nanowires with *V_z_* = 4Δ_0_. Other parameters are the same as those in Fig. 3.

Without loss of generality, we consider a sufficiently long *l*_0_ and *r*_0_ = 6ξ and μ = 1.2Δ_0_. Obviously, with or without the effective ZF, the two MZMs were always degenerated at *j* = 0 and *E* = 0 [see Fig. 3(a) for *V_z_* = 0 and Fig. 3(b) for *V_z_* = 4Δ_0_]. Meanwhile, the bottom and top MZMs are well separated [Fig. 3(c)–(e)]. Tracing the wave function of MZMs with increasing *V_z_*, the vortex MZM on the lower surface gradually becomes an edge mode [Fig. 3(c)–(e)]. Moreover, further comparing the energy spectrum with and without the effective ZF, we found that the ZF does yield extra in-gap states [Fig. 3(b)]. Notably, the energy difference between the MZMs and the first excited state gives the effective gap, which determines the upper limit of the ambient temperature and operating speed desired to manipulate the MZMs. Therefore, we plot the first excited state energy *E*_1_, indicating the effective gap, versus the radius of the cylindrical model *r*_0_ with a fixed wire length [Fig. 4(a)]. The excited energies become significantly quantized and *E*_1_ grows close to half of Δ_0_ as the radius *r*_0_ shrinks to below 10ξ [44]. In addition, a lower chemical potential |μ| yields a larger energy gap. We also plot the energy gap versus μ for different cylindrical radii *r*_0_ [Fig. 4(b)]. As |μ| moves farther away from the Dirac point of the topological surface states, the energy gap indeed reduces for any *r*_0_ case. Notably, even if the radius changes to *r*_0_ = 20ξ ≈ 100 nm, the energy of the first excited state still has 0.1Δ_0_ ≈ 0.18 meV.

By neglecting the bulk states, we analytically calculated the approximate function of the excited energy (see Sec. III within the online supplementary material for details)

(12) \begin{eqnarray*} \tilde{E}_1 \approx \frac{\pi }{\tilde{r}_0 ( 1+ \tilde{\mu }^2 )} \quad (\tilde{r} \gg 1), \end{eqnarray*}

with the rescaled $\tilde{E}_1 = E_1/\Delta _0$, $\tilde{\mu } = \mu /\Delta _0$, $\tilde{r}_0 = r_0/\xi$. This approximate function confirms the changing trend of *E*_1_, inversely proportional to *r*_0_ and μ^2^, in our iron-based superconducting nanowire system.

Notably, in the 2D vortex system, braiding MZMs can be achieved by tuning the coupling of the edge MZMs [31]. In that case, suppressing the coupling of edge chiral MZMs and vortex center MZMs requires increasing the distance between them, whereas ensuring a considerable energy gap between chiral MZMs and other edge states requires reducing the system size. In 2D topological SCs, these two contradictory conditions are difficult to reconcile because there is only one adjustable size parameter: the system radius. However, for iron-based superconducting nanowires, these two conditions correspond to two independently tunable parameters, nanowire length *l*_0_ and radius *r*_0_, respectively, and can thus be simultaneously satisfied. These reflect the unique advantages of the iron-based superconducting nanowire system.

## COUPLED TWO-EDGE MZMs

**Figure 5.**
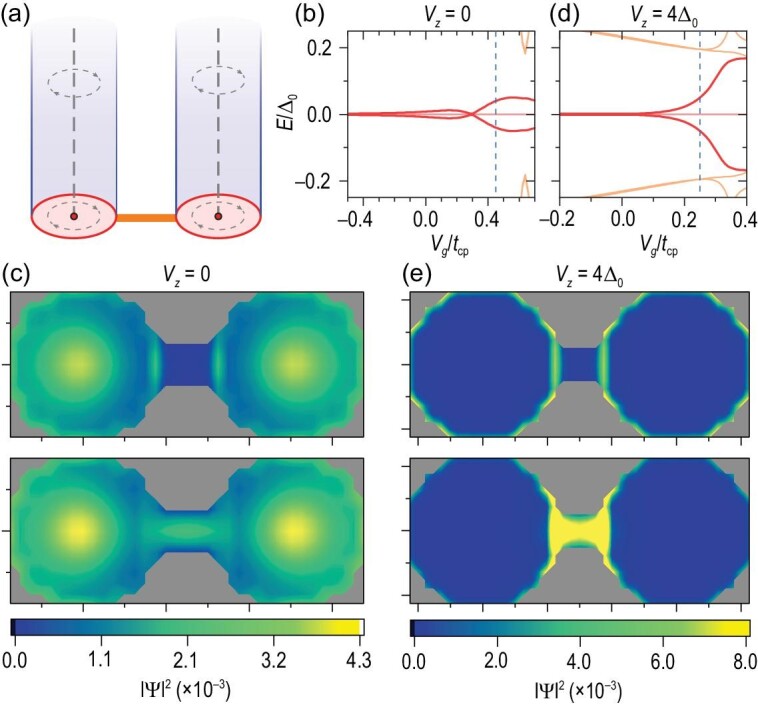
(a) Schematic of two coupled MZMs at the bottom of two parallel nanowires described by Equation (13). (b) Spectra of the two-nanowire system with radius *r*_0_ = 4ξ as a function of the potential on the connection *V_g_*. (c) Probability density of two decoupled (*V_g_* = −0.5*t*_cp_) and coupled (*V_g_* = 0.45*t*_cp_, corresponding to the blue dashed line in the spectra) Majorana vortex states at the bottom layer. (d) Spectra of the two-nanowire system with effective local ZFs at the bottom surface. (e) Probability density of two decoupled (*V_g_* = −0.5*t*_cp_) and coupled (*V_g_* = 0.25*t*_cp_, the blue dashed line in the spectra) edge Majorana states induced by the effective local ZFs. The nanowire parameters are the same as those in Fig. 1, expect that *M* = 4 meV and lattice constants ξ/2 (ξ/4) perpendicular (parallel) to the *z* direction.

The core for braiding edge MZMs is to control the coupling between different MZMs [31]. To verify the feasibility of such a scheme in the iron-based superconducting nanowires, we explore the coupling of two MZMs at the end of two wires [Fig. 5(a)], whose Hamiltonian takes the form

(13) \begin{eqnarray*} H_{\rm tot}= H_{\rm S} +H_{\rm N} +H_{\rm NS}, \end{eqnarray*}

where *H*_S_ denotes the iron-based SC system given in Equation (1). Because of the lack of rotational symmetry, we now use a 3D tight-binding model with a cubic lattice. In *H*_S_, we adjust some parameters to facilitate the calculation, without changing the topological property. The two-vortex superconducting order parameter is set as

(14) \begin{eqnarray*} \Delta (\boldsymbol {r}) = \Delta _0 \tanh \frac{r_1}{\xi } e^{i\varphi _1} \tanh \frac{r_2}{\xi } e^{i\varphi _2} , \end{eqnarray*}

where *r_i_* and ϕ_*i*_(*i* ∈ {1, 2}) denote the horizontal distances and azimuth angles measured from the vortex lines in the two nanowires, respectively. Any closed loop containing *n* vortex lines changes the phase of $\Delta (\boldsymbol {r})$ by 2*n*π. A gate-tunable Sm lead connects the two nanowires from the bottom edge. We simulate the lead as a square lattice

(15) \begin{eqnarray*} H_{\rm N} = t_{\rm cp}\hat{\tau }_z ( 4-2\cos {k_x}-2\cos {k_y} ) -V_g ,\\ \end{eqnarray*}

and attach it to the superconducting nanowires with

(16) \begin{eqnarray*} H_{\rm NS} = -t_{\rm cp} \sum _\alpha ( c_{{\rm N}\alpha }^\dagger \hat{\tau }_z c_{{\rm S}\bar{\alpha }} +{\rm H.c.} ) , \end{eqnarray*}

where $\bar{\alpha }$ denotes the sites at the nanowire edge attaching the ends α of the lead. The hopping strength is set as *t*_cp_ = 15 meV. In addition, *V_g_* denotes an adjustable on-site potential controlled by the gate voltage.

For the thin superconducting nanowires with diameters *d*_0_ = 8ξ ≈ 42 nm, the MZMs have finite distributions at the edges, as we mentioned in the section before last. Varying the potential *V_g_*, we plot the low-energy spectrum of the system [Fig. 5(b)]. For *V_g_* ≪ 0, where the Fermi level is far away from the energy band bottom in the connecting lead, the wave functions of two bottom MZMs are disconnected, as shown in the upper panel of Fig. 5(c). In addition, their energies keep zeros, degenerating with the two vortex center MZMs on the top surfaces. Adjusting the potential to *V_g_* = 0.45*t*_cp_, the two MZMs open a clear energy gap of about 0.04Δ_0_ ≈ 0.07 meV but still away from the excited states to avoid information leakage [Fig. 5(b)]. Meanwhile, parts of the two bottom MZMs penetrate the connecting lead and couple, as shown in the lower panel of Fig. 5(c). Moreover, the MZMs on the top are unaffected.

For the thicker superconducting nanowires, the weight of MZMs at the edges and the coupling by the connecting lead will be lower. However, we could enhance the coupling by adding an effective ZF *V_z_* = 4Δ_0_ at the bottom surface, as we discussed in the previous section. We now consider nanowires of diameters *d*_0_ = 10ξ with local effective ZFs as an example. The variations in the spectrum and MZM wave functions are shown in Fig. 5(d)–(e). Similarly, the two-edge MZMs can be isolated or connected under the gate *V_g_* adjustment. Although some in-gap interferential edge states are induced by the effective ZF, the energies of the coupled edge MZMs grow exponentially away from the degenerated zero-energy space as *V_g_* increases under the excited states.

## RESTRICTIONS ON THE NUMBER OF VORTICES

So far, we have assumed that each superconducting nanowire contains only one vortex. If there are two vortices with the same chirality in the nanowire, the nanowire diameter will limit the distance separating them, which will cause a finite repulsive potential and increase the system's free energy. Therefore, it is not surprising that repulsive interactions limit the number of vortices that penetrate the nanowire. The contribution of the vortices to the free energy, through their induced magnetic fields $\boldsymbol {h}(\boldsymbol {r})$, can be expressed as [45]

(17) \begin{eqnarray*} \Delta F = \frac{1}{8\pi } \int [\vert \boldsymbol {h}(\boldsymbol {r})\vert ^2 +\vert \boldsymbol {\nabla } \times \boldsymbol {h}(\boldsymbol {r})\vert ^2 ] d^3\boldsymbol {r}.\\ \end{eqnarray*}

When there is one vortex in the system with rotational symmetry along the *z* direction, the increased free energy is

(18) \begin{eqnarray*} \Delta F_1 = \frac{\Phi _0 L}{8\pi } h_1(\xi ), \end{eqnarray*}

where, in the range *r* ∈ (ξ, λ), the induced magnetic field takes the form

(19) \begin{eqnarray*} h_1(r) \approx \frac{\Phi _0}{2\pi \lambda ^2} \bigg ( \ln \frac{\lambda }{r} +0.12 \bigg ) \end{eqnarray*}

with λ the penetration depth of the magnetic field. When there are two vortices in the system, the induced magnetic field can be considered as the superposition of the magnetic field induced by each vortex [45]. Therefore, the increased free energy of the two vortices can be estimated as

(20) \begin{eqnarray*} \Delta F_2 = 2 \Delta F_1 + \frac{\Phi _0 L}{4\pi } h_1(2r_0) , \end{eqnarray*}

where the second term on the right-hand side is from the interaction of the two vortices separated by the maximum distance 2*r*_0_ inside the nanowire cross section.

Moreover, when the second vortex starts to appear in the system, the lower critical fields $H_{\rm c1}^{(n=1,\, 2)}$ satisfy the conditions [45] (see Sec. IV within the online supplementary material for details)

(21) \begin{eqnarray*} \Delta F_{1}= \frac{\int \boldsymbol {H}_{\rm c1}^{(n=1)} \cdot \boldsymbol {h}d^3 \boldsymbol {r}}{4\pi }=H_{\rm c1}^{(n=1)}\frac{\Phi _0 L}{4\pi } , \\ \end{eqnarray*}

(22) \begin{eqnarray*} \Delta F_2 -\Delta F_{1}=H_{\rm c1}^{(n=2)}\frac{\Phi _0 L}{4\pi } . \end{eqnarray*}

Substituting Equations (18) and (20) into Equations (21) and (22), we have

(23) \begin{eqnarray*} \delta H = H^{(n=2)}_{\rm c1} - H^{(n=1)}_{\rm c1} = H^{(n=1)}_{\rm c1} \frac{2h_1(2r_0)}{h_1(\xi )} .\\ \end{eqnarray*}

Clearly, the δ*H* is not negligible for small size, and it becomes larger as the distance between vortices becomes more restricted. For nanowires with 2*r*_0_ ∈ (ξ, λ), according to Equation (19), δ*H* can be estimated as

**Figure 6.**
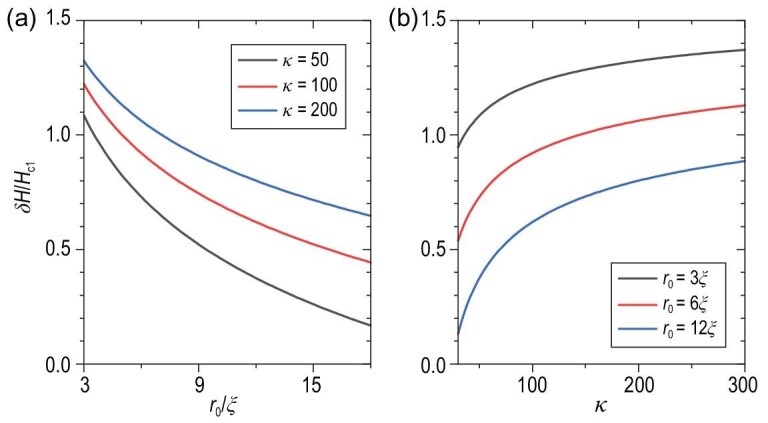
The estimated range of magnetic fields δ*H* when only a single vortex penetrates the system as a function of (a) the nanowire radius *r*_0_ and (b) the dimensionless Ginzburg–Landau parameter κ.

(24) \begin{eqnarray*} \frac{\delta H}{H^{(n=1)}_{\rm c1}} \approx 2\bigg [ 1- \frac{\ln 2\tilde{r}_{\rm 0}}{\ln \kappa } \bigg ] \end{eqnarray*}

with $\tilde{r}_0 = r_0/\xi$ and κ = λ/ξ as the dimensionless Ginzburg–Landau parameter. As shown in Fig. 6, materials with larger κ and made into thinner nanowires have larger δ*H*. Particularly for Fe(Se,Te), which satisfies κ ≈ 10^2^ [46] with *r*_0_ about dozens of ξ, δ*H* is of a similar magnitude as *H*_c1_. Thus, we can control the external magnetic field in the range (*H*_c1_, *H*_c1_ + δ*H*) to manufacture single vortex nanowires and prepare stable edge MZMs.

## CONCLUSION AND DISCUSSION

In this study, we propose an iron-based superconducting nanowire system to achieve controlled MZM. The finite radius of the nanowires limits the number of vortices penetrating them and stabilizes the ground-state degeneracy of the Majorana platform within a certain range of external magnetic fields. We found a size effect-induced TPT when the diameter of the iron-based superconducting nanowires is reduced to about πξ, approximately 20 nm for the Fe(Se,Te) nanowire, which yields a lower bound for the nanowire diameter. When the diameter is about (πξ, 4πξ), the MZMs have a limited distribution not only in the vortex center but also at the edges. For thick nanowires, edge MZMs can be obtained by inducing local effective ZFs, whereas the excitation energy of the disturbed edge states can be quantified by reducing the radius or decreasing the chemical potential. The edge MZMs in the nanowires can be connected in parallel by tunable Sm wires as a key step to achieving MZM manipulation.

As of now, the superconducting nanowires in the (100) direction have been fabricated [47–49]. The iron-based SCs have a strong TI band structure. Therefore, our theory is qualitatively applied to the (100)-direction nanowires; the details will be provided in future work. In most experimental studies, the fabrication control of impurities is necessary to tune the chemical potential but may lower the wire quality. A recent experimental study showed that applying strains in a suitable direction can tune the chemical potential in LiFeAs [50], which suggests a new technique of chemical potential control other than impurity doping. Thus, iron-based superconducting nanowires provide a promising platform for achieving controllable MZMs and studying MZM’s non-Abelian properties.

## Supplementary Material

nwac095_Supplemental_File
