# Supplementary material for: Controllable Majorana vortex states in iron-based superconducting nanowires
Source: Natl Sci Rev. 2022 May 17;9(9):nwac095. doi: 10.1093/nsr/nwac095 (PMC9521342; doi:10.1093/nsr/nwac095)
Supplement: nwac095_Supplemental_File [file nwac095_supplemental_file.pdf]

Supplemental Materials for "Controllable Majorana vortex states in iron-based superconducting nanowires"

Chuang Li,[1,2] Xun-Jiang Luo,[1,2] Li Chen,[1,2] Dong E. Liu,[3,*] Fu-Chun Zhang,[4,†] and Xin Liu[1,2,‡]

[1] *School of Physics and Institute for Quantum Science and Engineering, Huazhong University of Science and Technology, Wuhan, 430074, China*

[2] *Wuhan National High Magnetic Field Center and Hubei Key Laboratory of Gravitation and Quantum Physics, Wuhan, 430074, China*

[3] *State Key Laboratory of Low Dimensional Quantum Physics, Department of Physics, Tsinghua University, Beijing, 100084, China*

[4] *Kavli Institute for Theoretical Sciences, University of Chinese Academy of Sciences, Beijing 100190, China*

(Dated: April 11, 2022)

NUMERICALLY SOLVING THE FE(SE,TE) MODEL HAMILTONIAN

In Sec. II of the main text, we model the topological surface states in iron-based superconducting nanowires using a TI Hamiltonian which can be written as [1]

$$\begin{aligned} H_{\mathrm{TI}}(\mathbf{k}) =& A\hat{\sigma}_x\hat{\boldsymbol{s}}\cdot\mathbf{k}_\parallel + \hat{\sigma}_z(M - Bk_\parallel^2) \\ &+ \frac{A_3}{c}\hat{\sigma}_x\hat{s}_z \sin k_z c - \frac{B_3}{c^2}\hat{\sigma}_z(2 - 2\cos k_z c)\ , \end{aligned} \tag{1}$$

where we adopted long-wavelength approximation in the plane of $\mathbf{k}_\parallel = (k_x, k_y)$ for subsequent calculations. Here, c is the effective lattice constant along z-direction. $\hat{\boldsymbol{\sigma}}$ and $\hat{\boldsymbol{s}}$ are the Pauli matrices acting in the spin and orbital space respectively. A, A_3, B, B_3 and M are the material parameters in TI part. The coefficient A (A_3) equals the Fermi velocity of the topological surface states perpendicular (parallel) to z-direction and M indicates the half of the gap at Γ point. In order to provide more precise results for experiments, we use the parameters of an anisotropic strong TI model close to the practical band structure in Fe(Te,Se) [2], whose bulk dispersion is shown in Fig. 1, revealing a band inversion occurring at Z point and expected to generate topological surface states surrounding the nanowire's surface. Meanwhile, since $A_3 < \sqrt{2MB_3}$, the dispersion exhibits W-shape in k_z direction, which leads the bulk gap $E_{g,b} \approx 4.4$ meV to be smaller than the gap at high-symmetry point Z $E_{g,\mathrm{Z}} \approx 32.2$ meV. For a thick cylindrical model, it could be calculated that the

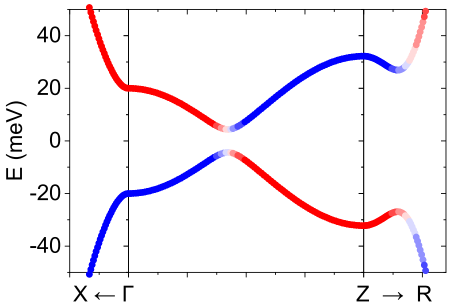

FIG. 1. The band structure of the TI part in the iron-based SC described by Eq. (1). The color maps orbital index $\langle\hat{\sigma}_z\rangle$, showing a band inversion at Z point.

vortex phase transition occurs at $\pm\mu_c = \pm A\sqrt{|E_{g,\mathrm{Z}}/B|} \approx \pm 30.6$ meV [3], that is, the MZMs exist in the range from $-\mu_c$ to μ_c, which could be verified by the topological invariant ν. While the radius-induced topological phase transition in thin nanowires has been discussed in Sec. II in the main text.

In the rest of this section, we will apply Bessel expansion on the simplified Fe(Se,Te) model to calculate the topological invariant ν, eigen-energies, and eigen-wavefunctions. We need to adopt k-space for z-direction (the direction of vortex line) when solve the topological invariant, while use full real space for eigen-states. Taking the former for example, we rewrite the TI bands part in the Fe(Se,Te) model Eq. (1) into real space cylindrical coordinate system as

$$\begin{aligned} H_{\mathrm{TI}}(r,\varphi,k_z) =& \hat{\sigma}_z \left[M + B \left(\partial_r^2 + \frac{1}{r}\partial_r + \frac{1}{r^2}\partial_\varphi^2 \right) \right] \\ & - iA\hat{\sigma}_x e^{-i\varphi\hat{s}_z} \left(\hat{s}_x \partial_r + \frac{1}{r}\hat{s}_y \partial_\varphi \right) + H_z(k_z) \end{aligned} \tag{2}$$

Inducing the SC term $\hat{\Delta}e^{i\varphi}$, we obtain the entire Hamiltonian H_{S} describing an iron-based SC vortex system. To simplify the following calculations, we could reduce the angular dimension using the continuous rotational symmetry $[\hat{J}_z, \mathcal{H}_{\mathrm{cyl}}] = 0$ in this cylindrical nanowire, with the z-component of total angular momentum $\hat{J}_z = \hat{L}_z + \hbar\hat{\tau}_z(\hat{s}_z - 1)/2$ where the orbital part $\hat{L}_z = -i\hbar\partial_\varphi$ and $\hat{\boldsymbol{\tau}}$ are the Pauli matrices in the particle-hole space. The wavefunctions take the form $\hat{U}_\varphi^{(j)}\Psi^{(j)}(r)$ where $\hat{U}_\varphi^{(j)} = \exp\{i[j - \hat{\tau}_z(\hat{s}_z - 1)/2]\varphi\}$ with the total magnetic quantum number $j \in \mathbb{Z}$ fulfilling the monodromy of wavefunctions and $\Psi^{(j)}(r)$ is a column vector independent of φ. So we could take the transform $\hat{U}_\varphi^{(j)}$ to reduce φ and get

$$\mathcal{H}_{\mathrm{nw}}^{(j)}(r,k_z) = \left(\hat{U}_\varphi^{(j)} \right)^{-1} H_{\mathrm{nw}}(r,\varphi,k_z) \hat{U}_\varphi^{(j)} \tag{3}$$

an effective Hamiltonian of r and k_z for any certain j.

The radial differential operators in $\mathcal{H}_{\mathrm{nw}}^{(j)}$ could be dealt with by the Bessel expansion method. Each component of the wavefunction $\Psi^{(j)}(r)$ with orbital angular quantum number m, corresponding the diagonal elements of $[j - \hat{\tau}_z(\hat{s}_z - 1)/2]$, can be expanded into a linear combination of a series of m-order Bessel functions. And in this Bessel representation, the matrix elements of the corresponding Hamiltonian $\mathcal{H}_{\mathrm{B}}^{(j)}$ can be obtained by

$$\left(\mathcal{H}_{\mathrm{B}}^{(j)} \right)_{p'q',pq} = \langle J_{q'}^{(m')} | \left(\mathcal{H}_{\mathrm{nw}}^{(j)} \right)_{p'p} | J_q^{(m)} \rangle \tag{4}$$

where $|J_q^{(m)}\rangle$ is the normalized Bessel function $J^{(m)}(\alpha_q^{(m)} r/r_0)/[r_0 J^{(m+1)}(\alpha_q^{(m)})/\sqrt{2}]$ with $\alpha_q^{(m)}$ the q-th zero (except the origin) of the m-order Bessel function. Discarding the high frequency oscillating Bessel functions corresponding large q which has little effect on the low-energy states, then we get the Hamiltonian matrix $\mathcal{H}_{\mathrm{B}}^{(j)}$ with finite size.

The topological region of this system can be obtained by regarding the superconducting vortex line as a quasi-1D system with particle-hole symmetry, and the corresponding $\mathbb{Z}_2$ topological invariant can be calculated as [4]

$$\nu = \text{sgn}\{\text{Pf}[\mathcal{H}^{(0)}_{\text{Mj}}(k_z = 0)]\}\text{sgn}\{\text{Pf}[\mathcal{H}^{(0)}_{\text{Mj}}(k_z = \pi)]\} \tag{5}$$

where $\mathcal{H}^{(j)}_{\text{Mj}}$ is the anti-symmetric Hamiltonian matrix under Majorana representation transformed from the $\mathcal{H}^{(j)}_{\text{B}}$.

If we keep the terms of z-direction in the tight-binding model, the spectrum and the eigen-wavefunctions of this cylindrical iron-based SC system can be calculated by diagonalizing $\mathcal{H}^{(j)}_{\text{B}}$.

In order to be close to the practical bands in the topological iron-based SC Fe(Te,Se) [2], in the calculations of the topological region and eigen-states of the single vortex system, we used an anisotropic TI model with parameter $M = 20$ meV, $B = -31$ meV·nm^2, $B_3 = 4.7$ meV·nm^2, $A = 30$ meV·nm, $A_3 = 2.7$ meV·nm and $c = 0.6$ nm. In the SC terms we used $\Delta_0 = 1.8$ meV, and hence the SC coherence length $\xi \approx 5.3$ nm while the characteristic length of MZMs $\xi_0 \approx 16.7$ nm is used as the unit length. In the 3D tight-binding model when we calculate the coupling of two MZMs, we used a cubic lattice with effective lattice constants $a = \xi/2$ in the horizontal plane and $c = \xi/4$ in z-direction. In order to adapt to the reduced energy bandwidth, we adjust the parameter $M = 4.0$ meV. The height $l_0 = 3\xi$ which is enough to isolate two MZMs at opposite ends of the vortex line. We used Kwant code [5] to construct the Hamiltonian and the PFAPACK library [6] to calculate the Pfaffian.

FINITE SIZE EFFECT OF TI'S LATERAL SURFACE STATES

In the radius-induced topological phase transition of the iron-based superconducting nanowire, the gap of the TI lateral surface states plays a key role and we will drive it in this section.

Let us force on the TI bands' Hamiltonian Eq. (1) at the band inversion point $k_z = \pi/c$

$$H_{\text{TI}} = A\hat{\sigma}_x \left(k_x\hat{s}_x + k_y\hat{s}_y\right) + (M' - Bk^2)\hat{\sigma}_z \tag{6}$$

where $M' = M - 4B_3/c^2$ and $M'B > 0$. In polar coordinate system, we could use the

rotational symmetry and rewrite the Hamiltonian as

$$\begin{aligned}\mathcal{H}_{\mathrm{TI}}^{(j)} = & -iA\hat{\sigma}_x\left(\hat{s}_x\partial_r + \frac{ij}{r}\hat{s}_y + \frac{1}{2r}\hat{s}_x\right) \\ & + \hat{\sigma}_z\left(M' + B\left[\partial_r^2 + \frac{1}{r}\partial_r - \frac{1}{r^2}\left(j - \frac{1}{2}\hat{s}_z\right)^2\right]\right)\end{aligned} \tag{7}$$

with $j \in \mathbb{Z} + 1/2$ now, and divide it into two blocks

$$\mathcal{H}_{\mathrm{TI}}^{(j)} = \mathcal{H}_1^{(j)} \otimes \mathcal{H}_2^{(j)} \tag{8}$$

$$\mathcal{H}_2^{(j)} = -\hat{s}_z\mathcal{H}_1^{(j)}\hat{s}_z \tag{9}$$

by transforming the representation from the basis $\begin{pmatrix} c_{a\uparrow} & c_{a\downarrow} & c_{b\uparrow} & c_{b\downarrow}\end{pmatrix}^T$ to $\left(\begin{pmatrix} c_{a\uparrow} & c_{b\downarrow}\end{pmatrix} \begin{pmatrix} c_{b\uparrow} & c_{a\downarrow}\end{pmatrix}\right)^T$. In the $j = 1/2$ sector,

$$\mathcal{H}_1^{(1/2)} = \begin{pmatrix} M' + B\left(\partial_r^2 + \frac{1}{r}\partial_r\right) & -iA\left(\partial_r + \frac{1}{r}\right) \\ -iA\partial_r & -M' - B\left(\partial_r^2 + \frac{1}{r}\partial_r - \frac{1}{r^2}\right)\end{pmatrix} \tag{10}$$

We define the new wavefunction $\bar{\Psi}(r) = \sqrt{r}\Psi(r)$ so that $2\pi\int|\bar{\Psi}(r)|^2 dr = 1$. Then, the corresponding Hamiltonian becomes

$$\bar{\mathcal{H}}_1^{(1/2)} = \begin{pmatrix} M' + B\left(\partial_r^2 + \frac{1}{4r^2}\right) & -iA\left(\partial_r + \frac{1}{2r}\right) \\ -iA\left(\partial_r - \frac{1}{2r}\right) & -M' - B\left(\partial_r^2 - \frac{3}{4r^2}\right)\end{pmatrix} \tag{11}$$

When the radius r_0 is very large, we anticipate there is a zero-energy edge state $\bar{\Psi}_0$ with $\langle 1/r\rangle \to 0$, and thus the Hamiltonian retains

$$\bar{\mathcal{H}}_0 = \hat{s}_z\left(M' + B\partial_r^2\right) - iA\hat{s}_x\partial_r \tag{12}$$

Assuming $\bar{\Psi}_0 = 1/\sqrt{2}\left(\begin{pmatrix} -i & \eta\end{pmatrix} \begin{pmatrix} 0 & 0\end{pmatrix}\right)^T \phi(r)$ $(\eta = \pm 1)$ which has $\hat{s}_y \oplus \hat{s}_y\bar{\Psi}_0 = \eta\bar{\Psi}_0$, then the Schrödinger equation gives

$$\left(M' + B\partial_r^2 + \eta A\partial_r\right)\phi(r) = 0 \tag{13}$$

Assuming $\phi(r) \propto e^{\lambda(r-r_0)}$, it becomes

$$B\lambda^2 + \eta A\lambda + M' = 0 \tag{14}$$

When $\eta = -1$, it has two roots with positive real part

$$\lambda_\pm = \frac{A \pm \sqrt{A^2 - 4M'B}}{2B} \tag{15}$$

Hence, $\phi(r) \propto e^{\lambda_+(r-r_0)} - e^{\lambda_-(r-r_0)}$ is the radius wavefunction of the edge states.

When the r_0 is small, the perturbation term

$$\bar{\mathcal{H}}' = \begin{pmatrix} B/4r^2 & -iA/2r \\ iA/2r & 3B/4r^2 \end{pmatrix} \tag{16}$$

can no longer be ignored. For simplicity, we use $\langle 1/r \rangle \approx 1/r_0$ for this exponentially decayed edge state $\bar{\Psi}_0$. Then we have

$$\langle \bar{\mathcal{H}}' \rangle = -\frac{A}{2r_0} + \frac{B}{2r_0^2} \tag{17}$$

Similarly, there is another edge state $\bar{\Psi}_0 \propto \left(\begin{pmatrix} 0 & 0 \end{pmatrix} \begin{pmatrix} -i & 1 \end{pmatrix} \right)^T$ with energy $-\langle \bar{\mathcal{H}}' \rangle$ we could obtain from $\mathcal{H}_2^{(j)}$.

Conclusively, as the radius r_0 decreases, the zero-energy TI's topological surface states on the side, which are proportional to $\begin{pmatrix} 1 & 0 & 0 & i \end{pmatrix}^T$ and $\begin{pmatrix} 0 & 1 & -i & 0 \end{pmatrix}^T$, open a gap $\pm \left(A/r_0 - B/r_0^2 \right)/2$ in $j = 1/2$ [7]. In the space of these two states and their hole part when the SC vortex is induced, we will find the size effect induced topological phase transition.

In addition, note that this energy gap and the phase transition is not due to the overlap between the TI surface states of the opposite surfaces. The energy gap due to the surface states overlapping is directly related to the surface states decay length l_0, which is determined by the bulk gap. According to the Fe(Se,Te) parameters, the band dispersion is W-shaped and the minimum bulk energy gap is at finite $k_m \approx \sqrt{M/B}$, as shown in Fig. 1. We could estimate the Fermi velocity $v_F = 2B \cdot k_m$, the bulk gap $E_{g,b} = A \cdot k_m$, and the surface states decay length $l_0 = v_F/E_{g,b} = 2B/A \approx 2$ (nm)[8]. However, the topological phase transition in the SC nanowire is at $r_c \approx 2\xi \approx 10$ (nm). This means that when the phase transition occurs, the surface state peaks at the nanowire edge and decays to zero before reaching $r = 0$, as shown in Fig. 2. Therefore, the phase transition here is not attributable to the overlap of the TI surface states.

ANALYTIC SOLUTION OF THE ZEEMAN FIELD INDUCED EDGE MZMS

In this section, we are going to solve the MZM localized at the boundary between superconducting topological surface and Zeeman field analytically, and estimate the energy gap between MZMs and excited states by perturbation to show the quantization of excited energies.

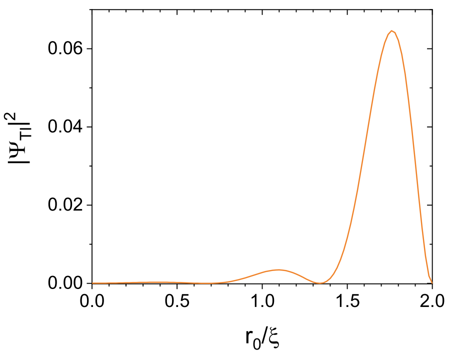

FIG. 2. The wavefunction of the lowest positive energy TI surface state at $r \approx r_c$, $j = 1/2$.

Let us focus on the southern hemisphere of the surface of a superconducting nanowire vortex system and map it to an infinite large 2D disk. On the whole disk, there are topological surface states from the TI component, whose effective Hamiltonian is equivalent to a Dirac cone

$$H_{\rm DC} = -iAe^{-i\varphi\hat{s}_z}\left(\hat{s}_x\partial_r + \frac{1}{r}\hat{s}_y\partial_\varphi\right) , \tag{18}$$

in polar coordinate system. The Zeeman field is mapped into $\hat{V}'_{\rm Z}(r) = -V_z\hat{s}_z\Theta(r_0 - r)$ exists in the center of the disk with a radius r_0 corresponding to the radius of the original nanowire. Outside the Zeeman field is the s-wave SC vortex region with the order parameter simplified as $\hat{\Delta}'(r)e^{i\varphi} = -i\hat{s}_y\Delta_0 e^{i\varphi}\Theta(r - r_0)$ since it's negligible in the center for a large Zeeman field $V_z \gg \Delta_0$. In summary, the Bogoliubov-de Gennes Hamiltonian of this disk could be written as

$$H_{\rm d} = \begin{pmatrix} H_{\rm DC}(\mathbf{r}) + \hat{V}'_{\rm Z} - \mu & \hat{\Delta}'e^{i\varphi} \\ \hat{\Delta}'^{\dagger}e^{-i\varphi} & -H^*_{\rm DC}(\mathbf{r}) - \hat{V}'_{\rm Z} + \mu \end{pmatrix} . \tag{19}$$

Again, taking advantage of the rotational symmetry $[\hat{J}_z, H_{\rm d}] = 0$ and using the method introduced in Appendix , we could get the Hamiltonian $\mathcal{H}^{(j)}_{\rm d}(r, \partial_r)$ for certain total magnetic quantum number j. Next we are going to look for the zero-energy wavefunction $\Psi_0(r) = \begin{pmatrix}\psi_{e\uparrow} & \psi_{e\downarrow} & \psi_{h\uparrow} & \psi_{h\downarrow}\end{pmatrix}^T$ at $j = 0$ in the Zeeman field and SC region respectively, which satisfies the Schrödinger equations

$$\mathcal{H}^{(0)}_{\rm d}\Psi_0(r) = 0 . \tag{20}$$

Firstly, let us consider the central Zeeman field region $r < r_0$. Since the SC term vanishes, the Hamiltonian $\mathcal{H}^{(j)}_{\rm d}$ could be divided into two blocks of the electron and the hole parts.

The general solutions without diverging at the origin $r = 0$ are the Bessel functions of imaginary argument

$$\begin{pmatrix} \psi_{e\uparrow} \\ \psi_{e\downarrow} \end{pmatrix} \propto \begin{pmatrix} I_0(\rho_1 r)\cos\frac{\theta}{2} \\ -iI_1(\rho_1 r)\sin\frac{\theta}{2} \end{pmatrix} , \begin{pmatrix} \psi_{h\uparrow} \\ \psi_{h\downarrow} \end{pmatrix} \propto \begin{pmatrix} I_0(\rho_1 r)\cos\frac{\theta}{2} \\ iI_{-1}(\rho_1 r)\sin\frac{\theta}{2} \end{pmatrix} , \tag{21}$$

with $\theta = 2\arctan[(V_z + \mu)/(V_z - \mu)]$ indicating the spin polarization direction and $\rho_1 = \sqrt{V_z^2 - \mu^2}/(A) \approx \sqrt{V_z^2 - \mu^2}/(\pi\Delta_0\xi)$ where ξ is the coherence length of SC. For $\rho_1 r \gg 1$, the approximate formula for m-order Bessel function of imaginary argument $I_m(\rho_1 r) \approx e^{\rho_1 r}/\sqrt{2\pi\rho_1 r}$ is independent of m. For $V_z \gg \mu$, $\theta \to \pi/2$ so that $\cos(\theta/2) = \sin(\theta/2)$. Then the zero-energy wavefunction on the Zeeman field side could be written as

$$\Psi_0(r) \propto \frac{1}{\sqrt{2}} \begin{pmatrix} C_1 & -iC_1 & C_2 & iC_2 \end{pmatrix}^T \sqrt{\frac{r_0}{r}} e^{\rho_1(r-r_0)} , \tag{22}$$

for the Zeeman field of sufficient large strength $V_z^2 \gg \Delta_0^2 + \mu^2$ and big size $r_0 \gg \xi_0$. C_1, C_2 are two undetermined constants.

Secondly, we will concentrate on the SC region where $r > r_0$ without Zeeman field. For algebraic simplicity, we first consider the case of $\mu = 0$, namely the Fermi level right on the Dirac point. The Hamiltonian in Eq. (20) could be block anti-diagonalized into two different spin parts, and each of them can be solved separately. For the spin-up component, its solution that converges at infinity is the MZM already proposed in the SC-TI interface model [9]

$$\begin{pmatrix} \psi_{e\uparrow} \\ \psi_{h\uparrow} \end{pmatrix} \propto \begin{pmatrix} 1 \\ -i \end{pmatrix} \phi(r) , \tag{23}$$

with

$$\phi(r) = \exp\left[-\frac{\Delta_0}{A}(r - r_0)\right] . \tag{24}$$

While for spin-down components, we could find another zero-energy solution $\begin{pmatrix} \psi_{e\downarrow} & \psi_{h\downarrow} \end{pmatrix}^T \propto \begin{pmatrix} 1 & i \end{pmatrix}^T r^{-1}\phi(r)$. It diverges at $r = 0$ but is still valid in this central Zeeman split model. Now, matching the wavefunctions at the boundary $r = r_0$ with ones in the central region, we get the coefficients $C_2 = -iC_1$ in Eq. (22), and the zero-energy state's wavefunction in SC region for $\mu = 0$ case is

$$\Psi_0(r) \propto \frac{1}{\sqrt{2}} \begin{pmatrix} 1 & -i\frac{r_0}{r} & -i & \frac{r_0}{r} \end{pmatrix}^T \phi(r) . \tag{25}$$

Then, we could pursuit for the low-energy excited energies with $m \neq 0$ for $\mu = 0$. The $m \neq 0$ part in Hamiltonian Eq. (20) can be view as a perturbation

$$\mathcal{H}'^{(j)} = \frac{jA}{r} \hat{\tau}_z \hat{s}_y \ , \tag{26}$$

and the excited energies could be calculated as $E_j = \langle \Psi_0 | H'^{(j)} | \Psi_0 \rangle / \langle \Psi_0 | \Psi_0 \rangle$. The zero-energy wavefunction has been solved in $\mu = 0$, but we could simplify it before the integration. Note that, leaving from $r = r_0$, the wavefunction in Zeeman field region is almost exponentially decay with length $\rho_1^{-1} \approx \xi_0 \Delta_0 / V_z$, while in SC region the decay length is approximate to ξ_0. Since $\Delta_0 \ll V_z$, the main part of integration is in the SC side, and we could ignore the Zeeman field part of the wavefunction in calculation. Furthermore, when $r_0 \gg \xi_0$, the inverse proportional factor appeared in spin-down components could be neglected. Under these approximations, we finally get

$$|\tilde{E}_j| \approx \frac{j}{\tilde{r}_0 + \frac{1}{2}} \ , \tag{27}$$

with the dimensionless quantities $\tilde{E}_j = E_j / \Delta_0$ and $\tilde{r}_0 = r_0 / \xi_0 = r_0 / (\pi \xi)$. Therefore, the gap between MZMs and the lowest excited energy E_1, which protects the MZMs' information, is approximately inversely proportional to the radius of Zeeman field r_0.

For $\mu \neq 0$ in SC region, refer to the wavefunctions we just solved, we could transform the Hamiltonian in Eq. (20) into the basis $\begin{pmatrix} c_\uparrow + ic_\uparrow^\dagger & c_\downarrow - ic_\downarrow^\dagger & c_\uparrow - ic_\uparrow^\dagger & c_\downarrow + ic_\downarrow^\dagger \end{pmatrix}^T$, then the solution that converges at infinity could be found via one of the submatrices in the block anti-diagonalized Hamiltonian

$$\begin{pmatrix} \psi_{e\uparrow} + i\psi_{h\uparrow} \\ \psi_{e\downarrow} - i\psi_{h\downarrow} \end{pmatrix} \propto \begin{pmatrix} C_3 J_0(\rho_2 r) + C_4 N_0(\rho_2 r) \\ -C_3 J_1(\rho_2 r) - C_4 N_1(\rho_2 r) \end{pmatrix} \phi(r) \ , \tag{28}$$

with $\rho_2 = \mu / A$ and J_m (N_m) is the Bessel (Neumann) function of n order. C_3, C_4 are two undetermined constants. Recalling the boundary conditions $\Psi_0(r_0) \propto \begin{pmatrix} 1 & -i & -i & 1 \end{pmatrix}^T$ for $\mu^2 \ll V_z^2 - \Delta_0^2$, we could approximate the zero-energy wavefunction as

$$\Psi_0(r) \propto \frac{1}{\sqrt{2}} \begin{pmatrix} \cos\left(\rho_2 (r - r_0) - \frac{\pi}{4}\right) \\ i \sin\left(\rho_2 (r - r_0) - \frac{\pi}{4}\right) \\ -i \cos\left(\rho_2 (r - r_0) - \frac{\pi}{4}\right) \\ -\sin\left(\rho_2 (r - r_0) - \frac{\pi}{4}\right) \end{pmatrix} \sqrt{\frac{r_0}{r}} \phi(r) \ . \tag{29}$$

Again, we could calculate the excited energies by perturbation theory for $\mu \neq 0$ cases. Here, in the integrand we have used an approximation to turn part of the probability density into a standard exponential shape for simplicity of calculation $(r_0/r)\exp[-2\Delta_0(r-r_0)/A] \approx \exp[(2\Delta_0/A + r_0^{-1})(r-r_0)]$. And finally the results of the excited energies are

$$|\tilde{E}_j| \approx \frac{j\left(\tilde{r}_0 + \frac{1}{2}\right)}{\left(\tilde{r}_0 + \frac{1}{2}\right)^2 + \left(\tilde{\mu}\tilde{r}_0\right)^2} \tag{30}$$

$$\approx \frac{j}{(1+\tilde{\mu}^2)\tilde{r}_0} , \tag{31}$$

with the dimensionless quantities $\tilde{E}_j = E_j/\Delta_0$, $\tilde{r}_0 = r_0/\xi_0 = r_0/(\pi\xi)$ and $\tilde{\mu} = \mu/\Delta_0$. The energy gap E_1 is inversely proportional to the Zeeman field's radius r_0 while approximately inversely proportional to the square of chemical potential μ. Thus, in order to get a large energy gap to reduce the interaction between MZMs and excited states, it is important to control the chemical potential close to the Dirac point in this system and make the cylinder slenderer.

VORTICES IN FINITE SIZE SYSTEMS

In this section, we are going to investigating the free energy of the superconducting nanowire vortex system, and prove that there is indeed a finite range of the external magnetic field that limits the vortices number to one.

Applying a external magnetic field H_{ext} on the superconducting nanowire in the length direction, the first vortex will penetrate the SC when H_{ext} is at the nanowire's lower critical field $H_{c1}^{(n=1)}$. And further more, we assume that the second vortex will appear at $H_{c1}^{(n=1)} + \delta H$ (we use H for magnetic field instead of Hamiltonian in this section). With the change of H_{ext}, the Gibbs free energy of the superconducting system is always continuous, and through that, we could relate H_{c1} and δH to the vortices' free energies [10].

In general, if there are n vortices penetrating the nanowire, each with a flux Φ, then the Gibbs free energy G_n can be written as

$$G_n = F_n - H_{\text{ext}} \frac{n\Phi L}{4\pi} . \tag{32}$$

Here, F_n is the Helmholtz free energy of the superconductor containing n vortices and L is the length of nanowire. The energy of n vortices is $\Delta F_n = F_n - F_0$. At $H_{\text{ext}} = H_{c1}^{(n=1)}$,

the continuity of Gibbs free energy requires $G_0 = G_1$, and this gives the energy of a single vortex

$$\Delta F_1 = H_{c1}^{(n=1)} \frac{\Phi L}{4\pi} \ . \tag{33}$$

At $H_{\rm ext} = H_{c1}^{(n=1)} + \delta H$, from $G_1 = G_2$ we obtain $\Delta F_2 - \Delta F_1 = (H_{c1}^{(n=1)} + \delta H)\Phi L/(4\pi)$. Here the energy of two vortices consists of $\Delta F_2 = 2\Delta F_1 + F_{\rm int}$ with $F_{\rm int}$ the interacting term. Then we have

$$F_{\rm int} = \delta H \frac{\Phi L}{4\pi} \ . \tag{34}$$

Therefore, there is a finite suitable magnetic field range δH as the positive $F_{\rm int}$.

Next we will estimate δH in a superconducting nanowire with a radius r_0 [10]. From the Ginzburg-Landau equation and Maxwell equation, we could derive the magnetic field distribution generated by a single vortex $h_1(r)$, which approximates to the 0-order Hankel function of imaginary argument

$$h_1(r) \approx h_0 \left(\ln \frac{\lambda}{r} + 0.12 \right) \ , \tag{35}$$

at $\xi < r < \lambda$ with λ the penetration depth of magnetic field and ξ the SC coherence length. h_0 is the characteristic magnetic field strength. And through this, we could calculate the single vortex energy

$$\Delta F_1 = \frac{\lambda^2 h_0}{4} h_1(\xi) \ , \tag{36}$$

and the interaction energy between two vortices $F_{\rm int} = \lambda^2 h_0 h_1(r_2)/2$, where $h_1(r_2)$ indicates the magnetic field distribution at the second vortex core generated by the first one. To minimize the repulsion between two vortices as well as the Gibbs free energy G_2, the distance between the two vortices should be maximized, namely $2r_0$ in the cross-section of the nanowire, then

$$F_{\rm int} = \frac{\lambda^2 h_0}{2} h_1(2r_0) \ . \tag{37}$$

Substituting them into Eq. (33) and Eq. (34), and according to the approximate shape of field $h_1(r)$, we get

$$\frac{\delta H}{H_{c1}} = \frac{F_{\rm int}}{\Delta F_1} = \frac{2h_1(2r_0)}{h_1(\xi)} \approx 2\left[1 - \frac{\ln 2\tilde{r}_0}{\ln \kappa}\right] \ , \tag{38}$$

where the diameter, $\tilde{r}_0 = r_0/\xi \in (1/2, \kappa/2)$ and the dimensionless Ginzburg-Landau parameter $\kappa = \lambda/\xi$.

---

* dongeliu@mail.tsinghua.edu.cn

† fuchun@ucas.ac.cn

‡ phyliuxin@hust.edu.cn

[1] C.-X. Liu, X.-L. Qi, H. Zhang, X. Dai, Z. Fang, and S.-C. Zhang, Phys. Rev. B **82**, 045122 (2010).

[2] P. Zhang, Z. Wang, X. Wu, K. Yaji, Y. Ishida, Y. Kohama, G. Dai, Y. Sun, C. Bareille, K. Kuroda, T. Kondo, K. Okazaki, K. Kindo, X. Wang, C. Jin, J. Hu, R. Thomale, K. Sumida, S. Wu, K. Miyamoto, T. Okuda, H. Ding, G. D. Gu, T. Tamegai, T. Kawakami, M. Sato, and S. Shin, Nature Physics **15**, 41 (2019).

[3] P. Hosur, P. Ghaemi, R. S. K. Mong, and A. Vishwanath, Phys. Rev. Lett. **107**, 097001 (2011).

[4] A. Y. Kitaev, Physics-Uspekhi **44**, 131 (2001).

[5] C. W. Groth, M. Wimmer, A. R. Akhmerov, and X. Waintal, New Journal of Physics **16**, 063065 (2014).

[6] M. Wimmer, ACM Trans. Math. Softw. **38**, 10.1145/2331130.2331138 (2012).

[7] M. Governale, B. Bhandari, F. Taddei, K.-I. Imura, and U. Zülicke, New Journal of Physics **22**, 063042 (2020).

[8] S.-Q. Shen, *Topological Insulators: Dirac Equation in Condensed Matters*, 1st ed., Springer Series in Solid-State Sciences 174 (Springer-Verlag Berlin Heidelberg, 2012).

[9] L. Fu and C. L. Kane, Phys. Rev. Lett. **100**, 096407 (2008).

[10] M. Tinkham, *Introduction to Superconductivity: Second Edition*, Dover Books on Physics (Dover Publications, 2004).
